# Supplementary material for: Cancer-associated snaR-A noncoding RNA interacts with core splicing machinery and disrupts processing of mRNA subpopulations
Source: Nat Commun. 2025 Nov 25;16:10460. doi: 10.1038/s41467-025-65448-x (PMC12647172; doi:10.1038/s41467-025-65448-x)
Supplement: Supplementary file 1 — Supplementary Information [file 41467_2025_65448_MOESM1_ESM.pdf]

Supplementary Figures

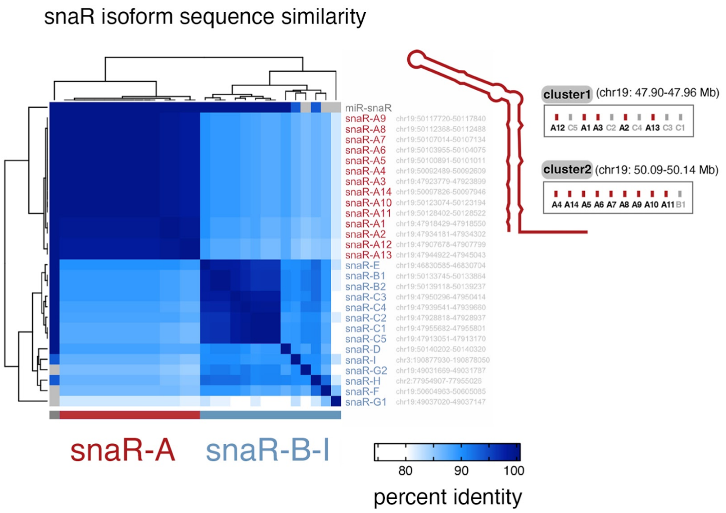

Supplementary Figure 1. Sequence similarity of snaR-A, -B, -C, -D, -E, -F, -G, -H, and -I isoforms. Hierarchical clustering of individual snaR isoform sequences on the basis of sequence similarity (percent identity), with sequences corresponding to snaR isoform A (snaR-A; red) notably distinct from those corresponding to snaR isoforms B-I (blue). Chromosome and genomic intervals correspond to GRCh38. Inset highlights the individual positions of snaR-A genes in clusters 1 and 2 on chr19, mapped to isoform number. (Related to Figure 1) Source data are provided as a Source Data file.

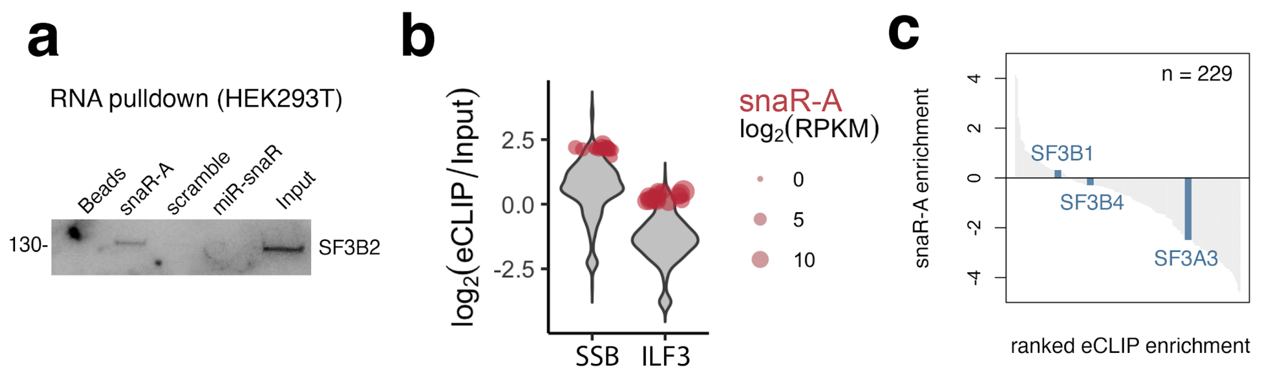

Supplementary Figure 2. snaR-A interaction with SF3B2 and enrichment across La (SSB) and ILF3 eCLIP experiments, compared against Pol III-transcribed genes. (a) Immunoblot analysis of SF3B2 protein following biotin-snaR-A pull-down, compared to beads, biotin-scramble RNA, and biotin-miR-snaR RNA. **(b)** Violin plots represent the eCLIP enrichment distributions of all Pol III-transcribed genes for La (left) and ILF3 (right). snaR-A genes, highlighted in red, are among the top enriched RNA species in both experiments **(c)** Analogous eCLIP survey bar plot, highlighting snaR-A enrichment in experiments for U2 snRNP proteins SF3A3, SF3B1, and SF3B4. (Related to Figure 1) Source data are provided as a Source Data file.

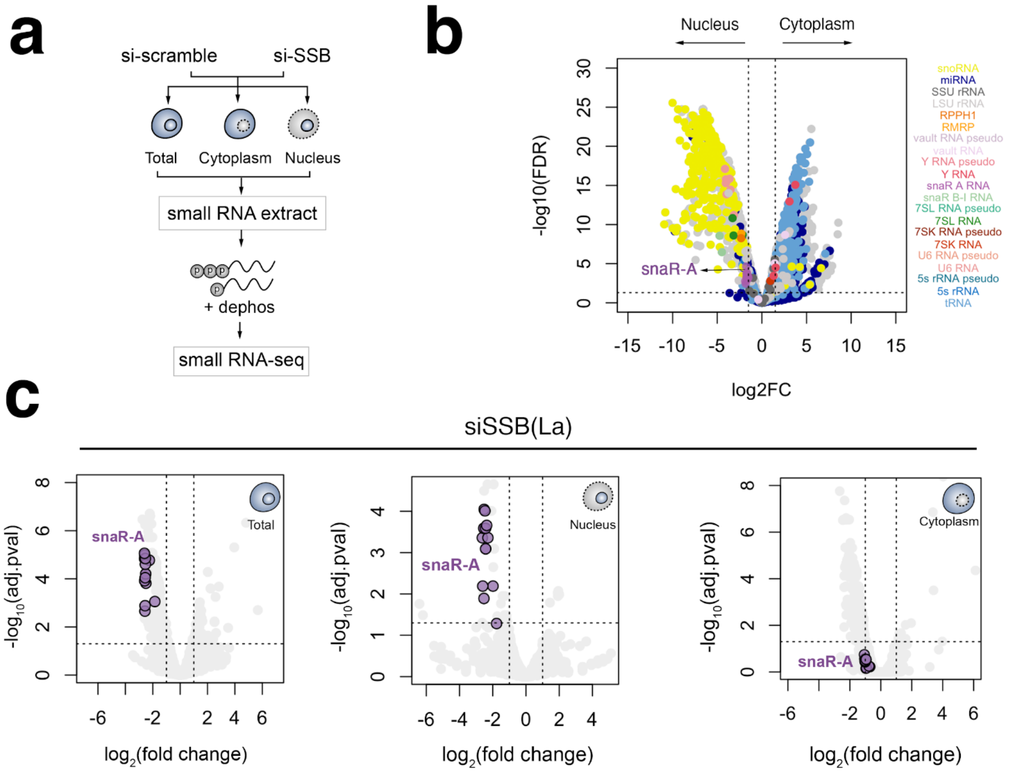

Supplementary Figure 3. Subcellular fractionation small RNA-seq captures differential enrichment of snaR-A within the nucleus. (a) Illustration of workflow of small RNA-seq in HEK293T after SSB(La) knockdown (n = 3 biological replicates) **(b)** Volcano plot of small RNA nucleus/cytoplasmic enrichment **(c)** Volcano plot visualization of differentially expressed small RNA genes upon SSB(La) knockdown in the total, nuclear, and cytoplasmic cell fraction. snaR-A is highlighted in purple. Source data are provided as a Source Data file.

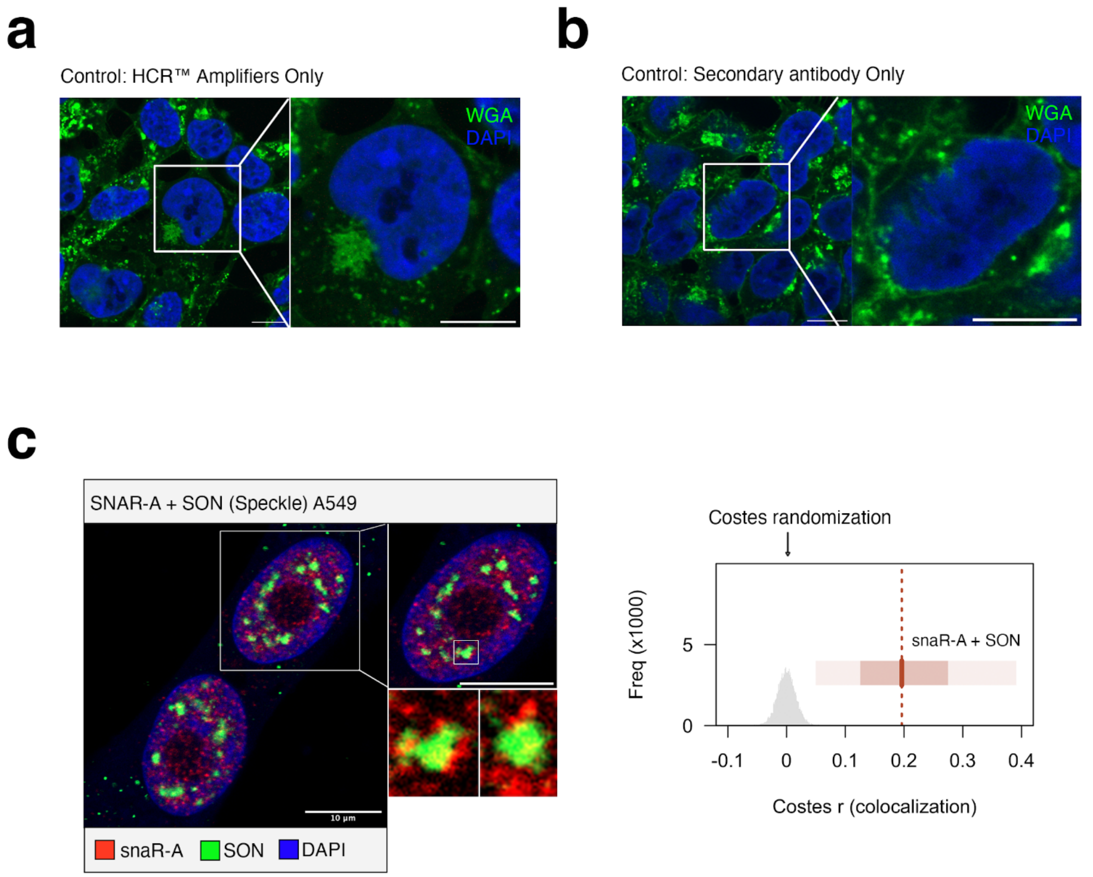

Supplementary Figure 4. snaR-A localizes to subnuclear foci that are associated with splicing bodies (speckles) in A549 cells (a) Negative control for HCR-RNA-FISH with HCR amplifiers only in HEK293T cells, staining for DAPI (blue) and wheat germ agglutinin (WGA; membrane) (green) Scale bar, 10 μm. **(b)** Negative control for HCR-RNA-FISH with secondary antibody only in HEK293T cells, staining for DAPI (blue) and wheat germ agglutinin (WGA; membrane) (green) Scale bar, 10 μm. **(c)** HCR-RNA-FISH detection of snaR-A in A549, co-stained for SON (nuclear speckles) and DAPI. (left) Quantitative analysis of in situ feature co-localization. Observed correlation distributions are compared against Costes randomization-based null hypotheses (right) Scale bars, 10 μm. Box plots show the median (center), the 25th and 75th percentiles. Light shade present minimum and maximum values. Source data are provided as a Source Data file.

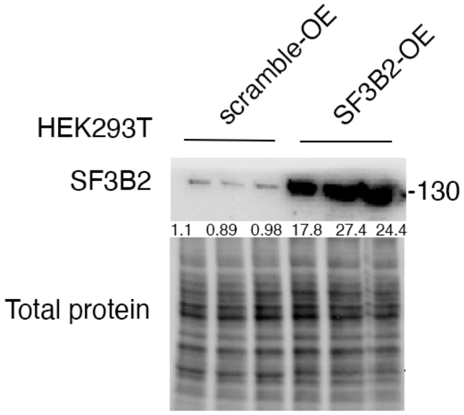

Supplementary Figure 5. SF3B2 overexpression in HEK293T (a) Immunoblots and quantification of protein levels of SF3B2 following SF3B2 overexpression. (n = 3 biological replicates) Source data are provided as a Source Data file.

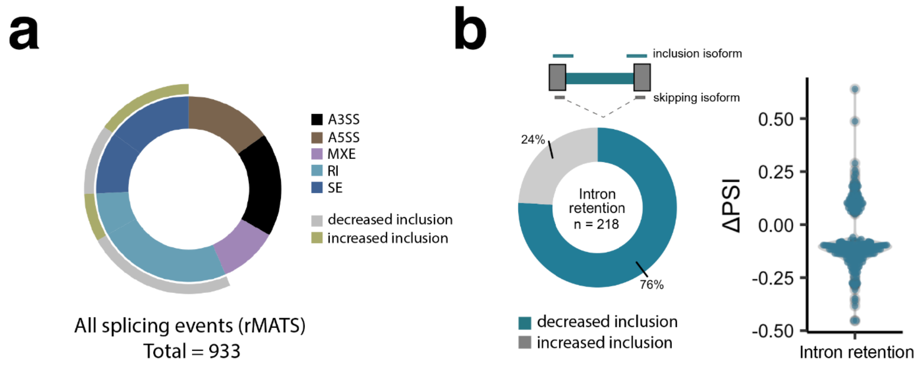

Supplementary Figure 6. Alternative splicing events identified by rMATS following snaR-A depletion. (a) Illustration of breakdown of 933 significantly alternatively spliced events into various event categories. A3SS: Alternative 3′ splice site (n = 169), A5SS: Alternative 5′ splice site (n = 141), MXE: Mutually exclusive exon (n = 96), RI: Retained intron (n = 287), SE: Skipped exon (n = 240). **(b)** Breakdown of 287 significant RI events identified by rMATS, with 76% decreased intron retention and 21% increased intron retention. Source data are provided as a Source Data file.

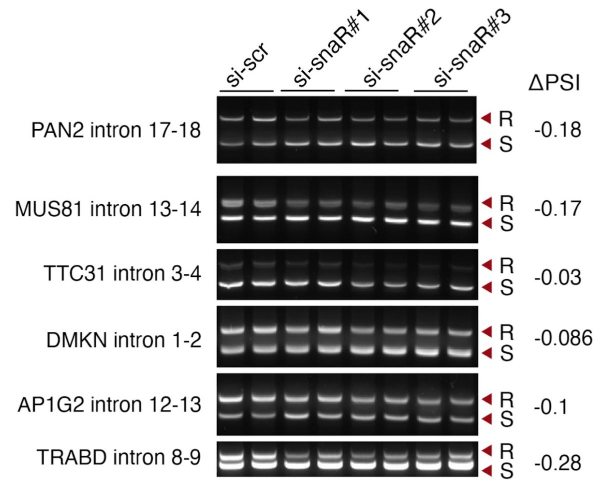

Supplementary Figure 7. PCR analysis and validation of differential intron retention splicing events identified by rMATS following snaR-A depletion. PCR analysis of intron retention (IR) levels corresponding to specific introns determined to be significantly downregulated following snaR-A depletion. Introns were tested for several independent transcripts (PAN2, MUS81, TTC31, DMKN, AP1G2, and TRABD). Arrows indicate retained (R) and spliced (S) introns. Delta PSI (Percentage Spliced In) values indicate the change in IR levels following snaR-A depletion, negative values indicate a lower proportion of transcripts with the intron retained (i.e. decreased intron retention). (n = 2 biological replicates) Source data are provided as a Source Data file.

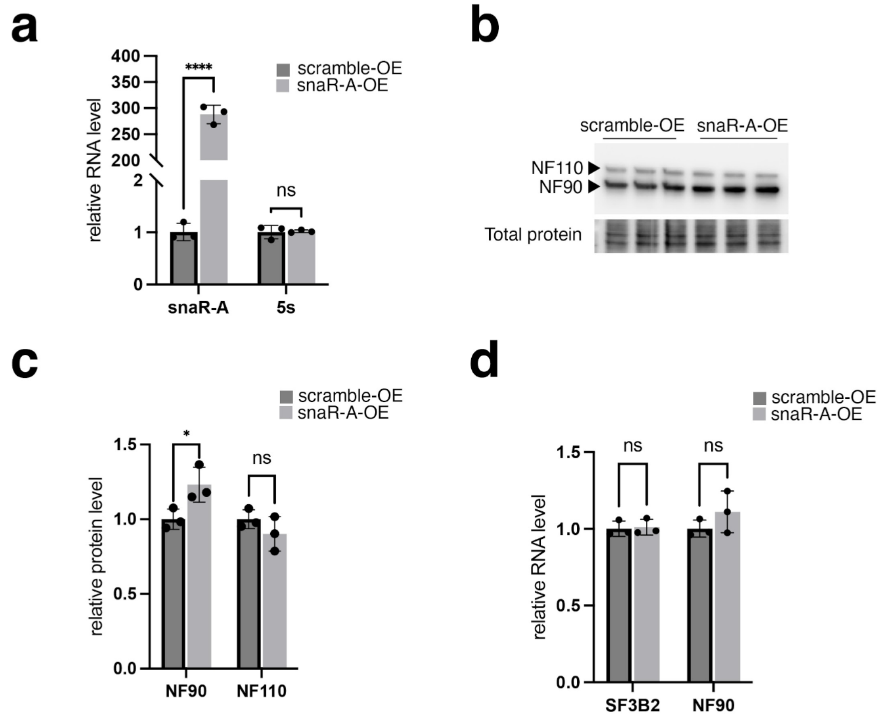

Supplementary Figure 8. **Overexpression of snaR-A does not disrupt ILF3 isoforms NF90 and NF110, nor SF3B2 RNA levels.** (**a**) RT-qPCR analysis of snaR-A and 5s rRNA levels following snaR-A overexpression indicating successful overexpression of snaR-A RNA. (**b,c**) Immunoblots (b) and quantification (c) of protein levels of NF110 and N90 following snaR-A overexpression. (**d**) RT-qPCR analysis of SF3B2 and NF90 mRNA levels following snaR-A overexpression. Data are presented as mean ± standard deviation (SD) from the indicated number of independent samples. Biological replicates = 3; two-group comparison analyzed with t-test; * $p \leq 0.05$; ** $p \leq 0.01$; *** $p \leq 0.001$; **** $p \leq 0.0001$. Source data are provided as a Source Data file.

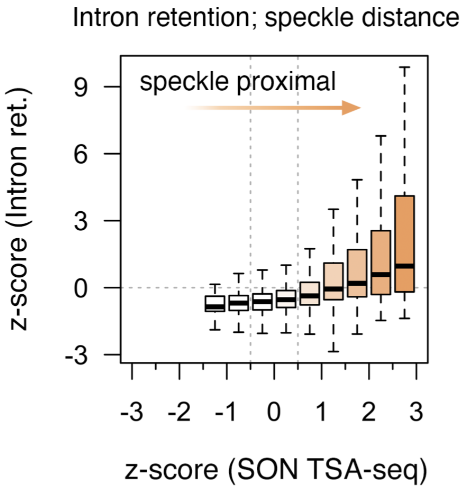

Supplementary Figure 9. **Transcript-level intron retention (IR) scores are elevated in mRNAs derived from speckle-proximal genes.** Distributions of intron retention levels as a function of nuclear speckle distance (SON TSA-seq score). Box plots show the median (center), the 25th and 75th percentiles (bounds of the box), and whiskers extending to values within 1.5× the interquartile range. (n = 28 [-1.5, -1], n = 944 [-1,-0.5], n = 2427 [-0.5,0], n = 2190 [0,0.5], n = 1743 [0.5,1], n = 1474 [1,1.5], n = 1240 [1.5,2], n = 525 [2,2.5], n = 52 [2.5,3]) Source data are provided as a Source Data file.

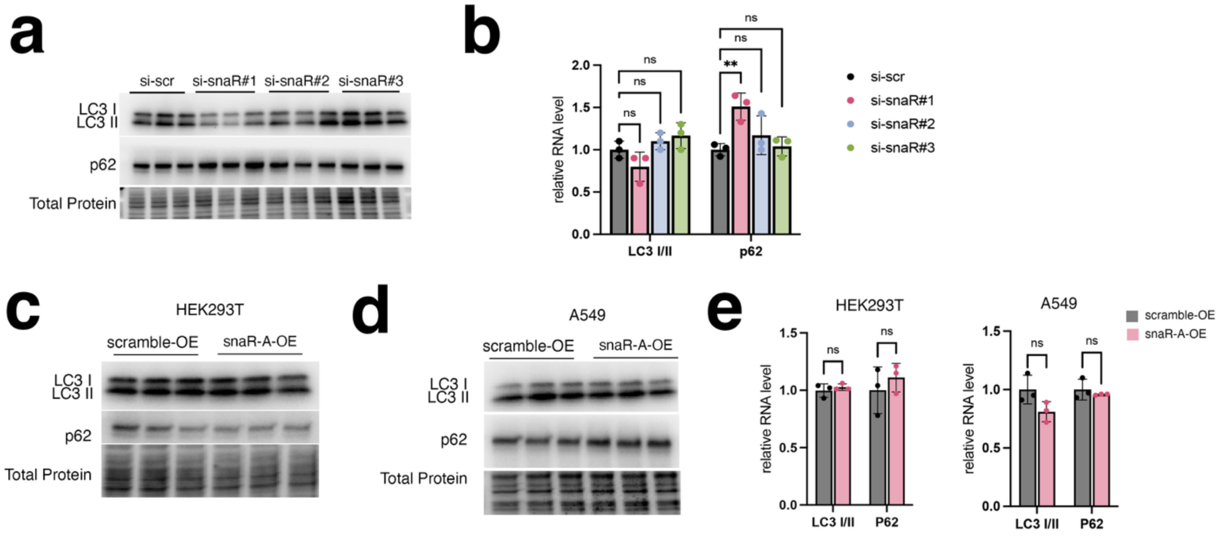

Supplementary Figure 10. Overexpression and knockdown of snaR-A do not affect autophagy marker expression levels. (**a**,**b**) Immunoblots (a) and quantification (b) of protein levels of autophagy marker LC3 and p62 following snaR-A knockdown in HEK293T cell. (**c-e**) Immunoblots (c,d) and quantification (e) of protein levels of autophagy marker LC3 and p62 following overexpression in HEK293T and A549. Protein levels are normalized to total protein level. Data are presented as mean ± standard deviation (SD) from the indicated number of independent samples. Biological replicates = 3; two-group comparison analyzed with t-test; multi-group comparison analyzed with ANOVA; * $p \leq 0.05$; ** $p \leq 0.01$; *** $p \leq 0.001$; **** $p \leq 0.0001$. Source data are provided as a Source Data file.

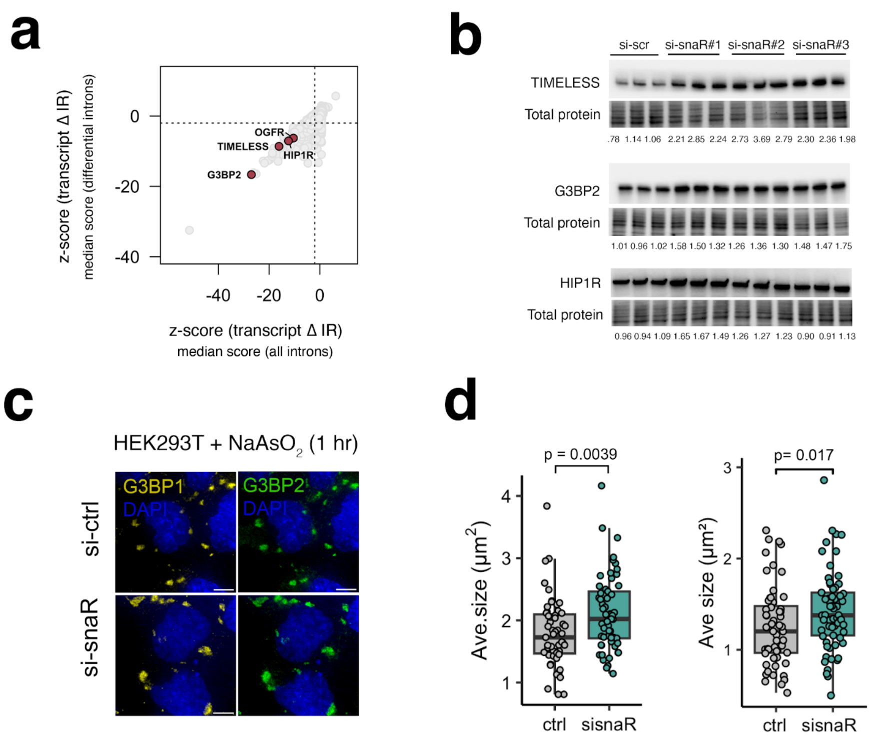

Supplementary Figure 11. Depletion of snaR-A increases stress granule size, concomitant with increased G3BP2 protein abundance. (a) Analysis of the transcript-wide survey results including ghost (“all”) intron scores (x-axis) versus the same survey with ghost introns omitted (y-axis) identified 136 unique genes with significant differential IR levels following snaR-A depletion (adjusted p-val < 0.05 in both approaches) **(b)** Immunoblot analysis and quantification of protein levels corresponding to genes with differential IR levels (TIMELESS, G3BP2, and HIP1R; highlighted in panel a) (n = 3 Biological replicates) **(c)** Immunofluorescence microscopy of stress granule marker G3BP1 (yellow) and G3BP2 (green) following snaR-A knockdown and NaAsO2 treatment (1hr) to induce stress granule. Images are maximum-intensity z projected for a 1 μm section. Scale bars, 5 μm **(d)** Quantifications of average stress granule size using G3BP1 as an indicator for stress granules across two biological replicates. Box plots show the median (center), the 25th and 75th percentiles (bounds of the box), and whiskers extending to values within 1.5× the interquartile range. Two-group comparison analyzed with Wilcox test. Source data are provided as a Source Data file.

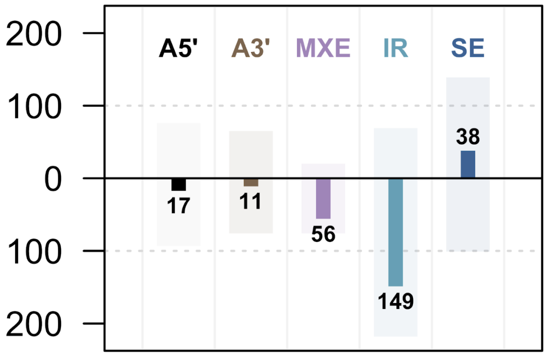

Supplementary Figure 12. rMATS analysis of alternative splicing events following snaR-A depletion. Barplots indicate the number of significant events that increase (above 0) or decrease (below 0) following snaR-A depletion and the directionality bias when taking such events into account. A5SS = alternative 5' splice site; A3SS = alternative 3' splice site; MXE = mutually exclusive exon; IR = retained intron; SE = skipped exon. Source data are provided as a Source Data file.

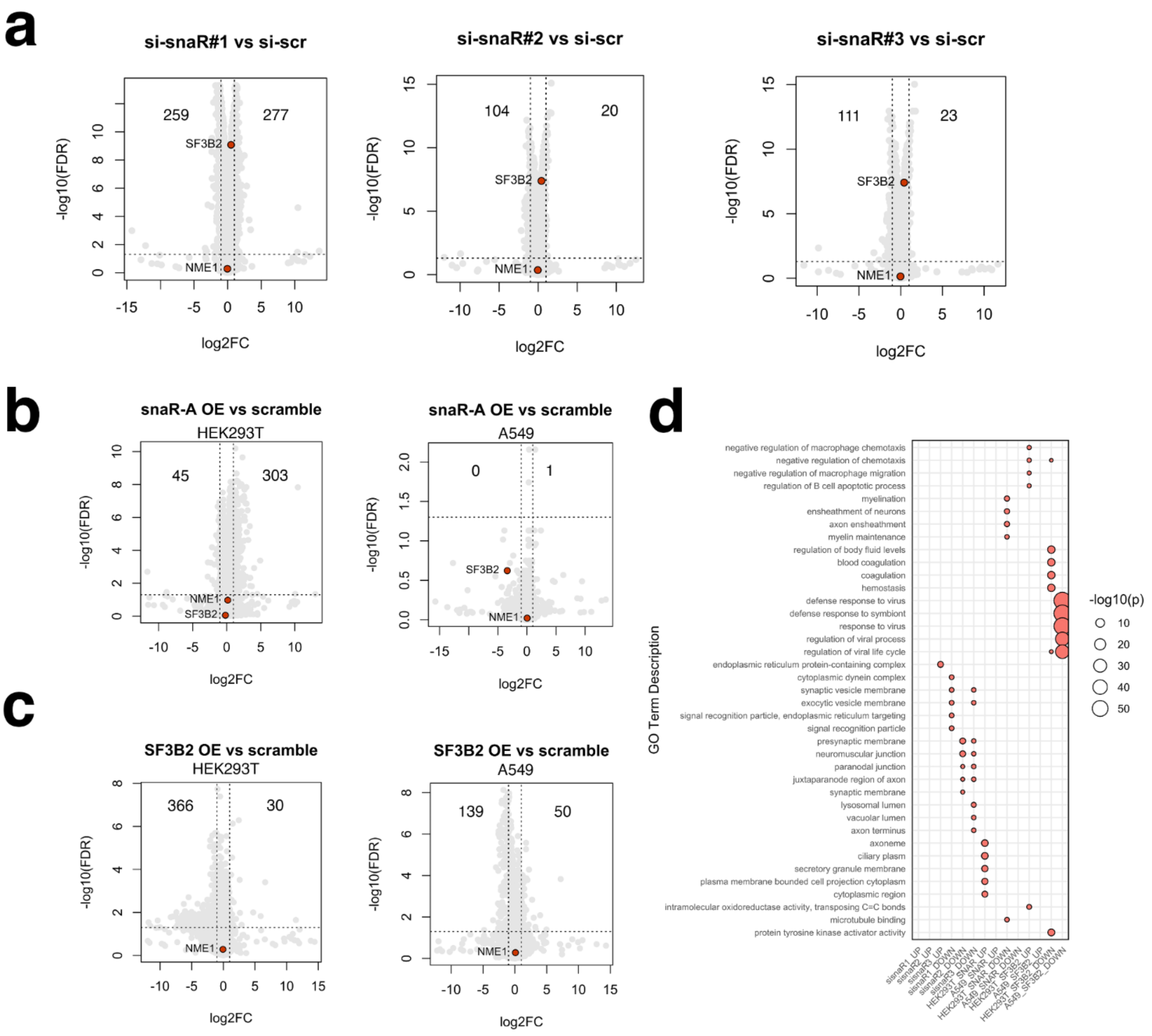

Supplementary Figure 13. Differential gene expression analysis following snaR-A depletion, overexpression and SF3B2 overexpression. (a-c) Volcano plot visualization of differentially expressed gene upon snaR-A knockdown using three different siRNA (a), snaR-A overexpression in HEK293T and A549 (b), SF3B2 in HEK293T and A549 (c). NME1 and SF3B2 are highlighted in red. Significance calculated using edgeR two-sided exactTest function, Benjamini–Hochberg corrected p-value. **(d)** Gene ontology (GO) enrichment analysis on transcripts identified in panel a-c. Significance is determined using FDR < 0.05, logFC > 1 or < -1. Source data are provided as a Source Data file.

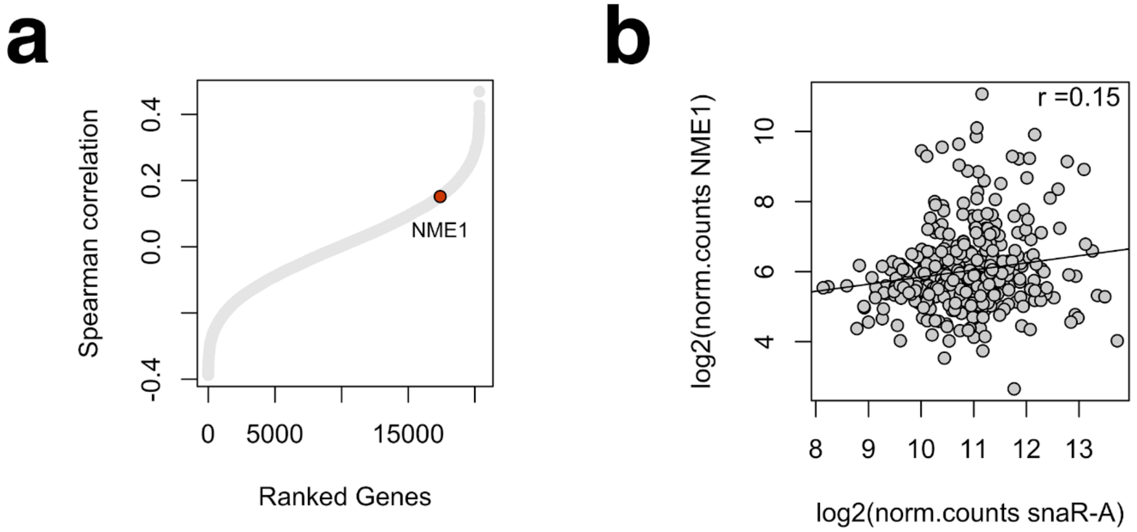

Supplementary Figure 14. NME1 expression does not negatively correlate with snaR-A gene activity in primary tumors. (a) S-plot of Spearman correlation between snaR-A gene activity and mRNA expression levels across primary tumors. Highlighting NME1. **(b)** Scatter plot of $\log_2$(normalized.counts) for snaR-A gene activity and NME1 expression level. Spearman correlation r= 0.15. Source data are provided as a Source Data file.

Supplementary Table

Supplementary Table 1. siRNA sequences for snaR-A knockdown

Name	Sequence
si-scramble	5'-UUCUCCGAACGUGUCACGUtt-3'
si-snaR-A#1	5'-CCACAUGGGUCGGAAAAAAtt-3'
si-snaR-A#2	5'-GGUCCACAUGGGUCGGAAAtt-3'
si-snaR-A#3	5'-UGGUCCACAUGGGUCGGAAtt-3'

Supplementary Table 2. Taqman™ probe sequences for RT-qPCR

Target	Assay ID	Context sequence
RNU18	001204	
Z30	001092	
snaR-A	CTZTET6	CCTCACGGAGGCGGGGGTTCCAGGGCACGAGTTCGAGGCCAGCCTGGTCCACATGGGTCG
RNU6	001973	
GAPDH	Hs02786624_g1	
ACTB	Hs99999903_m1	
SF3B2	Hs00199190_m1	
NF90	Hs01128122_g1	

Supplementary Table 3. Antibody information

Target	Cat number	Dilution
La (SSB)	sc-80656 (Santa Cruz Biotechnology)	1:1000
SF3A1	15858-1-AP (proteintech)	1:1000
SF3A3	12070-1-AP (proteintech)	1:1000
SF3B4	10482-1-AP (proteintech)	1:1000
SF3B2	A5875 (ABclonal)	1:1000
MCRIP2	20808-1-AP (proteintech)	1:1000
OGFR	11177-1-AP (proteintech)	1:1000
TIMELESS	14421-1-AP (proteintech)	1:1000
HIP1R	16814-1-AP (proteintech)	1:1000
ILF3	19887-1-AP (proteintech)	1:1000
MTA1	30545-1-AP (proteintech)	1:1000
AUP1	13726-1-AP (proteintech)	1:500

Supplementary Table 4. RNA probe sequences for RNA pull-down

Name	Sequence
Biotin_snaR-A	/5Biosg/rCrC rGrGrA rGrCrC rArUrU rGrUrG rGrCrU rCrArG rGrCrC rGrGrU rUrGrC rGrCrC rUrGrC rCrCrU rCrGrG rGrCrC rCrUrC rArCrG rGrArG rGrCrG rGrGrG rGrUrU rCrCrA rGrGrG rCrArC rGrArG rUrUrC rGrArG rGrCrC rArGrC rCrUrG rGrUrC rCrArC rArUrG rGrGrU rCrGrG rArArA rArArA rGrGrA rUrUrU rUrUrU rU
Biotin_scramble	/5Biosg/rGrA rUrArG rUrGrC rGrUrA rGrGrA rGrGrU rUrGrC rUrGrG rArCrC rGrUrG rGrCrC rUrGrG rArGrU rCrUrU rCrCrG rCrGrC rUrGrG rCrGrU rCrGrC rGrGrA rCrCrU rGrUrC rUrArG rGrUrC rGrGrU rCrCrG rCrCrU rArUrG rUrCrA rGrGrU rArArG rCrArG rGrArC rArArC rUrCrA rCrCrG rArGrC rGrUrG rCrCrA rCrGrC rG
Biontin_miR-snaR	/5Biosg/rArG rCrCrU rGrGrU rCrCrA rCrArU rGrGrG rUrCrG rGrA
Biotin_scramble_miR-snaR	/5Biosg/rGrU rUrGrG rCrCrG rGrArU rGrCrA rArCrU rCrGrA rCrG

Supplementary Table 5. Primer sequences for Intron-Exon Junction PCR

Name	Sequence
MTA1-5-6-F	AAT GGA GAA CCC GGA AAT GGT G
MTA1-5-6-R	TCC AGG TAG GAC TTG AGC GAC T
MCRIP2-3-4-F	GGA GAA TGT CCG CTT TGT GTC C
MCRIP2-3-4-R	TCC TCT CCA CGT ACT GCA CAG G
MCRIP2-4-5-F	CCT GTG CAG TAC GTG GAG AGG A
MCRIP2-4-5-R	AGG AAC AGC TGG TGA TTC TCG C
OGFR-2-3-F	GGA CAT GTG TAG GTA TCG GCA C
OGFR-2-3-R	GCA GGA AGC GGA TCT CAT TTC T
PAN2-17-18-F	AAG AAC GTC TGG CTT CCT TTC T
PAN2-17-18-R	TCT CCA ACT TTG ATG TGA GCC A
MUS81-13-14-F	TGC TCA CTC CTC ACC TTC AGT G
MUS81-13-14-R	GGT GCT GTA TCG ATC CAC CAG G
TTC31-3-4-F	TAG TGG ATT TTC TTC GAC GGC T
TTC31-3-4-R	ACT CTT GGG GAG GAT ACA GGA A
DMKN-1-2-F	CAT GCT CGG AAT AAC TTC CTG C
DMKN-1-2-R	CCC GGG TTT ATG TCC ATT TCC T
AP1G2-12-13-F	TGG CAC ATA GAC ACC ATC CTG C
AP1G2-12-13-R	GCG CAC AGA GTA GGC ATG TAG C
TRABD-8-9-F	GAA GGA CCT ACT GGA GCA GAT G
TRABD-8-9-R	CAC GTC ATG ATC TCC TGG ATG T
