# Supplementary material for: Cancer-associated snaR-A noncoding RNA interacts with core splicing machinery and disrupts processing of mRNA subpopulations
Source: Nat Commun. 2025 Nov 25;16:10460. doi: 10.1038/s41467-025-65448-x (PMC12647172; doi:10.1038/s41467-025-65448-x)
Supplement: Supplementary file 2 — Description of Additional Supplementary Files [file 41467_2025_65448_MOESM2_ESM.pdf]

Description of Additional Supplementary Files

Supplementary Data 1. Liquid Chromatography-Tandem Mass Spectrometry analysis of snaR-A pulldown, scramble pulldown, and beads-only control
