# Supplementary material for: Cancer-associated snaR-A noncoding RNA interacts with core splicing machinery and disrupts processing of mRNA subpopulations
Source: Nat Commun. 2025 Nov 25;16:10460. doi: 10.1038/s41467-025-65448-x (PMC12647172; doi:10.1038/s41467-025-65448-x)
Supplement: Supplementary file 4 — Reporting Summary [file 41467_2025_65448_MOESM4_ESM.pdf]

nature portfolio

Corresponding author(s): Kevin Van Bortle

Last updated by author(s): Jul 28, 2025

Reporting Summary

Nature Portfolio wishes to improve the reproducibility of the work that we publish. This form provides structure for consistency and transparency in reporting. For further information on Nature Portfolio policies, see our Editorial Policies and the Editorial Policy Checklist.

Statistics

For all statistical analyses, confirm that the following items are present in the figure legend, table legend, main text, or Methods section.

n/a	Confirmed	
☐	☒	The exact sample size (*n*) for each experimental group/condition, given as a discrete number and unit of measurement
☐	☒	A statement on whether measurements were taken from distinct samples or whether the same sample was measured repeatedly
☐	☒	The statistical test(s) used AND whether they are one- or two-sided<br>*Only common tests should be described solely by name; describe more complex techniques in the Methods section.*
☐	☒	A description of all covariates tested
☐	☒	A description of any assumptions or corrections, such as tests of normality and adjustment for multiple comparisons
☒	☐	A full description of the statistical parameters including central tendency (e.g. means) or other basic estimates (e.g. regression coefficient) AND variation (e.g. standard deviation) or associated estimates of uncertainty (e.g. confidence intervals)
☐	☒	For null hypothesis testing, the test statistic (e.g. *F*, *t*, *r*) with confidence intervals, effect sizes, degrees of freedom and *P* value noted<br>*Give P values as exact values whenever suitable.*
☒	☐	For Bayesian analysis, information on the choice of priors and Markov chain Monte Carlo settings
☒	☐	For hierarchical and complex designs, identification of the appropriate level for tests and full reporting of outcomes
☐	☒	Estimates of effect sizes (e.g. Cohen's *d*, Pearson's *r*), indicating how they were calculated

Our web collection on statistics for biologists contains articles on many of the points above.

Software and code

Policy information about availability of computer code

Data collection	-Genome coordinates information used to generate statistical parameters included in the regression model is collected using R package: biomaRt v3.19<br>-Statistic parameters in the logistic regression including G/C content, transcript length, median intron length, 3'UTR length, 5'UTR length are calculated by custom code<br>-Predicted branch point score is generated using open source software BPP, branch point prediction<br>-Predicted G-quadruplex density of introns is generated using open source software G4Hunter and custom code<br>-Predicted Splice site strength is calculated using online web tool: MaxEntScan

Data analysis	TCGA data is analyzed using open source software: survival v3.6-4<br>Custom code for analyzing U2 Residency is available at: https://github.com/VanBortleLab/U2snRNP_ResidencyScore<br>eCLIP-seq and RNA -seq data is analyzed using open source software:<br>trimglore v0.6.5<br>bowtie2 v2.5.3<br>Salmon v1.5.2<br>samtools v1.9<br>bedtools v2.28.0<br>IRFinder v1.3.1<br>rMATs-turbo v4.1.1<br>R package: R V 4.1.2, edge R v3.19

For manuscripts utilizing custom algorithms or software that are central to the research but not yet described in published literature, software must be made available to editors and reviewers. We strongly encourage code deposition in a community repository (e.g. GitHub). See the Nature Portfolio guidelines for submitting code & software for further information.

Data

Policy information about availability of data

All manuscripts must include a data availability statement. This statement should provide the following information, where applicable:

- Accession codes, unique identifiers, or web links for publicly available datasets
- A description of any restrictions on data availability
- For clinical datasets or third party data, please ensure that the statement adheres to our policy

The RNA-seq data generated for this study are available through the NCBI Gene Expression Omnibus with accession number GSE271057, GSE286577 and GSE293548

The Cancer Genome Atlas (TCGA) ATAC-seq
-Genomic Data Commons Data Portal:https://portal.gdc.cancer.gov/

ENCODE eCLIP-seq
-ENCODE database : https://www.encodeproject.org/

SON TSA-seq
-4D Nucleome:https://www.4dnucleome.org/

Research involving human participants, their data, or biological material

Policy information about studies with human participants or human data. See also policy information about sex, gender (identity/presentation), and sexual orientation and race, ethnicity and racism.

Reporting on sex and gender	*Use the terms sex (biological attribute) and gender (shaped by social and cultural circumstances) carefully in order to avoid confusing both terms. Indicate if findings apply to only one sex or gender; describe whether sex and gender were considered in study design; whether sex and/or gender was determined based on self-reporting or assigned and methods used.*<br>*Provide in the source data disaggregated sex and gender data, where this information has been collected, and if consent has been obtained for sharing of individual-level data; provide overall numbers in this Reporting Summary. Please state if this information has not been collected.*<br>*Report sex- and gender-based analyses where performed, justify reasons for lack of sex- and gender-based analysis.*
Reporting on race, ethnicity, or other socially relevant groupings	*Please specify the socially constructed or socially relevant categorization variable(s) used in your manuscript and explain why they were used. Please note that such variables should not be used as proxies for other socially constructed/relevant variables (for example, race or ethnicity should not be used as a proxy for socioeconomic status).*<br>*Provide clear definitions of the relevant terms used, how they were provided (by the participants/respondents, the researchers, or third parties), and the method(s) used to classify people into the different categories (e.g. self-report, census or administrative data, social media data, etc.)*<br>*Please provide details about how you controlled for confounding variables in your analyses.*
Population characteristics	*Describe the covariate-relevant population characteristics of the human research participants (e.g. age, genotypic information, past and current diagnosis and treatment categories). If you filled out the behavioural & social sciences study design questions and have nothing to add here, write "See above."*
Recruitment	*Describe how participants were recruited. Outline any potential self-selection bias or other biases that may be present and how these are likely to impact results.*
Ethics oversight	*Identify the organization(s) that approved the study protocol.*

Note that full information on the approval of the study protocol must also be provided in the manuscript.

Field-specific reporting

Please select the one below that is the best fit for your research. If you are not sure, read the appropriate sections before making your selection.

- [x] Life sciences
- [ ] Behavioural & social sciences
- [ ] Ecological, evolutionary & environmental sciences

For a reference copy of the document with all sections, see nature.com/documents/nr-reporting-summary-flat.pdf

Life sciences study design

All studies must disclose on these points even when the disclosure is negative.

Sample size	Experiments assessing statistical significance were performed in biological duplicates, triplicates, or greater, following established protocols from previous publications, the required material amounts, and generally accepted practices
Data exclusions	No data was excluded from analysis
Replication	All experiments described in this manuscript were independently repeated with two or more biological replicates, and all replication attempts were successful.
Randomization	Sample allocation was randomized with respect to treatment conditions, and experiments for control and treatment groups were performed in parallel and analyzed together.
Blinding	Blinding was not performed due to use of non-subjective means of quantification and experiments on cell cultures without human/animal subjectivity

Reporting for specific materials, systems and methods

We require information from authors about some types of materials, experimental systems and methods used in many studies. Here, indicate whether each material, system or method listed is relevant to your study. If you are not sure if a list item applies to your research, read the appropriate section before selecting a response.

Materials & experimental systems

n/a	Involved in the study	
[ ]	[x]	Antibodies
[ ]	[x]	Eukaryotic cell lines
[x]	[ ]	Palaeontology and archaeology
[x]	[ ]	Animals and other organisms
[x]	[ ]	Clinical data
[x]	[ ]	Dual use research of concern
[x]	[ ]	Plants

Methods

n/a	Involved in the study	
[x]	[ ]	ChIP-seq
[x]	[ ]	Flow cytometry
[x]	[ ]	MRI-based neuroimaging

Antibodies

Antibodies used

La (SSB) sc-80656 (Santa Cruz Biotechnology) 1:1000
SF3A1 15858-1-AP (proteintech) 1:1000
SF3A3 12070-1-AP (proteintech) 1:1000
SF3B4 10482-1-AP (proteintech) 1:1000
SF3B2 A5875 (ABclonal) 1:1000
MCRIP2 20808-1-AP (proteintech) 1:1000
OGFR 11177-1-AP (proteintech) 1:1000
TIMELESS 14421-1-AP (proteintech) 1:1000
HIP1R 16814-1-AP (proteintech) 1:1000
ILF3 19887-1-AP (proteintech) 1:1000
MTA1 30545-1-AP (proteintech) 1:1000
AUP1 13726-1-AP (proteintech) 1:500

Validation

Anti-La (SSB) sc-80656 (Santa Cruz Biotechnology) is validated in Nat Commun. 9: 3284
Anti-SF3A1 15858-1-AP (proteintech) is validated in DOI: 10.1016/j.celrep.2015.03.048
Anti-SF3A3 12070-1-AP (proteintech) is validated in DOI: 10.3390/ijms232214174
Anti-SF3B4 10482-1-AP (proteintech) is validated in DOI: 10.1016/j.chembiol.2018.01.016
Anti-SF3B2 A5875 (ABclonal) is validated in https://doi.org/10.1016/j.bbrc.2022.05.046
Anti-MCRIP2 20808-1-AP (proteintech) is validated in DOI: 10.3390/biom5031441
Anti-OGFR 11177-1-AP (proteintech) is validated in DOI: 10.7150/ijbs.69655

Anti-TIMELESS 14421-1-AP (proteintech) is validated in DOI: 10.1016/j.canlet.2017.05.022
Anti-HIP1R 16814-1-AP (proteintech) is validated in DOI: 10.1016/j.cell.2021.07.020
Anti-ILF3 19887-1-AP (proteintech) is validated in DOI: 10.1016/j.cell.2021.02.008
Anti-MTA1 30545-1-AP (proteintech) is validated by manufacture by immunoblot using human samples
Anti-AUP1 13726-1-AP (proteintech) is validated in DOI: 10.1091/mbc.E16-07-0483

Eukaryotic cell lines

Policy information about cell lines and Sex and Gender in Research

Cell line source(s)	THP-1, A549 and HEK293T was obtained from ATCC
Authentication	THP-1, A549 and HEK293T cells were authenticated by ATCC, including STR analysis, COI analysis and morphology
Mycoplasma contamination	THP-1, A549 and HEK293T were confirmed to be mycoplasma negative prior to experiments
Commonly misidentified lines (See ICLAC register)	No cell lines registered on ICLAC were used in the study

Plants

Seed stocks	*Report on the source of all seed stocks or other plant material used. If applicable, state the seed stock centre and catalogue number. If plant specimens were collected from the field, describe the collection location, date and sampling procedures.*
Novel plant genotypes	*Describe the methods by which all novel plant genotypes were produced. This includes those generated by transgenic approaches, gene editing, chemical/radiation-based mutagenesis and hybridization. For transgenic lines, describe the transformation method, the number of independent lines analyzed and the generation upon which experiments were performed. For gene-edited lines, describe the editor used, the endogenous sequence targeted for editing, the targeting guide RNA sequence (if applicable) and how the editor was applied.*
Authentication	*Describe any authentication procedures for each seed stock used or novel genotype generated. Describe any experiments used to assess the effect of a mutation and, where applicable, how potential secondary effects (e.g. second site T-DNA insertions, mosiacism, off-target gene editing) were examined.*
